# Heat-Inactivation of Human Serum Destroys C1 Inhibitor, Pro-motes Immune Complex Formation, and Improves Human T Cell Function

**DOI:** 10.3390/ijms22052646

**Published:** 2021-03-05

**Authors:** Matthias A. Fante, Sonja-Maria Decking, Christina Bruss, Stephan Schreml, Peter J. Siska, Marina Kreutz, Kathrin Renner

**Affiliations:** 1 Department of Internal Medicine III, University Medical Center Regensburg, 93053 Regensburg, Germany; Matthias.Fante@ukr.de (M.A.F.); Sonja-Maria.Decking@ukr.de (S.-M.D.); Christina.Bruss@ukr.de (C.B.); peter.siska@ukr.de (P.J.S.); marina.kreutz@ukr.de (M.K.); 2 Regensburg Center for Interventional Immunology, 93053 Regensburg, Germany; 3 Department of Dermatology, University Medical Center Regensburg, 93053 Regensburg, Germany; Stephan.Schreml@ukr.de

**Keywords:** T cell activation, complement, serum heat-inactivation, C1 inhibitor, immune complexes, cytokines

## Abstract

Heat-inactivation of sera is used to reduce possible disturbing effects of complement factors in cell-culture experiments, but it is controversially discussed whether this procedure is appropriate or could be neglected. Here, we report a strong impact of heat-inactivation of human sera on the activation and effector functions of human CD4+ T cells. While T cells cultured with native sera were characterized by a higher proliferation rate and higher expression of CD28, heat-inactivated sera shaped T cells towards on-blast formation, higher cytokine secretion (interferon γ, tumor necrosis factor, and interleukin-17), stronger CD69 and PD-1 expression, and increased metabolic activity. Heat-inactivated sera contained reduced amounts of complement factors and regulators like C1 inhibitor, but increased concentrations of circulating immune complexes. Substitution of C1 inhibitor reduced the beneficial effect of heat-inactivation in terms of cytokine release, whereas surface-molecule expression was affected by the addition of complex forming anti-C1q antibody. Our data clearly demonstrate a beneficial effect of heat-inactivation of human sera for T cell experiments but indicate that beside complement regulators and immune complexes other components might be relevant. Beyond that, this study further underpins the strong impact of the complement system on T cell function.

## 1. Introduction

The complement system is an ancient proteolytic host defense cascade comprising more than 30 factors, receptors and regulators that circulate in the serum and are bound to cell surfaces. Initiation of the complement cascade by recognition of pathogen-associated patterns (PAMPs) and binding to immune complexes proceeds via three distinct pathways—the classical, the lectin, and the alternative pathways [1,2,3,4]. Upon activation, complement conducts innate responses against foreign invaders and supports attraction of immune effector cells, phagocytosis, and formation of the membrane attack complex (MAC) resulting in cell lysis [5,6,7].

Since the 1970s there has been increasing evidence that complement factors also take on key modulatory functions in adaptive immunity, which was early demonstrated in B cells [8,9]. Substantial literature illustrates that binding of complement-coated antigens to complement receptors 1 (CR1, CD35) and 2 (CR2, CD21) exerts co-stimulatory signals during B cell activation [10,11,12], acts as an endogenous molecular adjuvant lowering the threshold for B cell activation, and prevents the expansion of auto-reactive B cell clones [13,14,15].

More recent findings have demonstrated an impact of complement factors on T cell immunity. Complement influences T cell activation on two levels—directly via surface complement receptors and regulators and indirectly via the alteration of antigen-presenting cell (APC) function. Studies with C3-deficient mice reveal an impaired anti-viral CD4+ T cell response in an influenza infection model [16] and humans with C3-deficiency lack an adequate Th1 response and thus suffer from recurrent infections [17]. Professional APCs carry the entire repertoire of complement surface receptors which facilitate phagocytosis of opsonized antigens and initiate maturation [1,18]. In combination with Toll-like receptors (TLRs), anaphylatoxins C3a and C5a, as well as C3b-coated antigens, shape APCs towards inducing a Th1 effector response by upregulation of MHC II, co-stimulatory molecules (e.g., CD86), and secretion of IL-12 [19,20,21]. Other complement factors have ambivalent regulatory effects, since C1q induces a pro- or anti-inflammatory T cell response depending on the environmental context [22,23]. Remarkably, also findings obtained in experiments on allogeneic organ rejection show that predominantly, complement not derived from the liver but generated by immune cells themselves mediates modulatory effects on APCs and T cells in an auto- and paracrine manner [24,25,26].

T cells produce, and store intracellularly, C3, which is continuously cleaved by the protease cathepsin L (CTSL) [27]. During T cell receptor (TCR) and CD28 signaling, these cleavage products are released and bind to the C3a receptor (C3aR) and the complement regulator CD46 on the cell surface [27]. The binding of C3a leads to an upregulation of IL-12 receptors and an activation of the PI3K-Akt-mTORC1 pathway, which improves a Th1 response and IFNγ secretion [28,29]. CD46 is a ubiquitously expressed, C3b-binding complement regulator [30,31,32]. Intracellular signaling activates the mitogen-activated protein (MAP) kinase cascade and promotes cell cycle progression, expression of IL-2 receptor family members and energy-delivering processes such as anaerobic glycolysis and mitochondrial respiration [33,34,35,36,37,38]. What is more, Jiang et al., have shown that binding of C1q-coated immune complexes to T cells improves activation, and the concomitant IFNγ and TNF secretion even without TCR signaling [39], via specific C1q-binding sites [40], while on the other hand C1q-mediated anti-proliferative signaling has been reported [41,42]. To sum up, complement tightly links innate and adaptive immunity, has a tremendous impact on T cell activation and, in particular, promotes a robust Th1 response.

In order to prevent negative effects of complement factors, heat-inactivation of serum (56 °C for 30 min) is a common but controversially discussed step in cell culture protocols as besides complement inactivation [43] unintended effects such as the aggregation of serum proteins [44] and degradation of growth factors, vitamins, and amino acids cannot be ruled out. Heat-inactivation shows variable effects on proliferation of different cell types in vitro. While the proportion of bovine blastocysts is increased under heat-inactivated conditions [45], another comparison of 11 different cell lines predominantly shows no advantage of heat-inactivation on proliferation (*Art To Science*, Vol. 15, No. 1, 1996, HiClone). In the late 1990s Leshem et al., demonstrated, that heat-inactivation of fetal calf serum has no impact on proliferation, IL-2 secretion, or cytotoxic activity of murine lymphocytes [46]. To the best of our knowledge, data on the impact of serum heat-inactivation on human T cells are missing. Here, we investigated the influence of heat-inactivation of human sera on the function of cultured CD4+ T cells.

## 2. Results

### 2.1. Heat-Inactivation of Human Serum Has Significant Impact on Complement Factors, Regulators, and Immune Complexes

**Figure 1:**
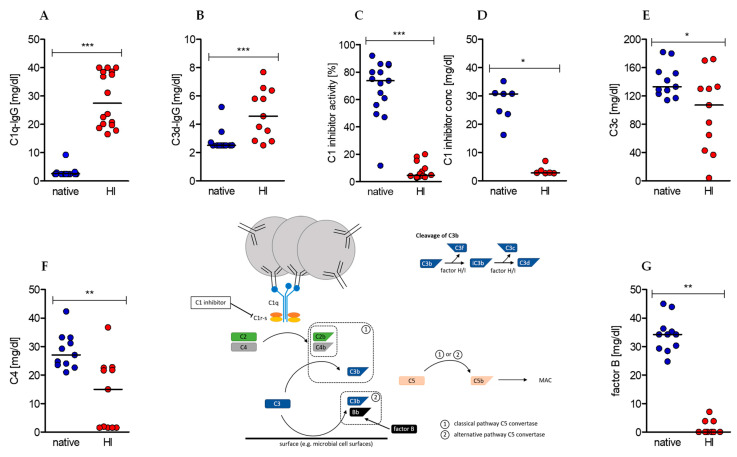
Effects of serum heat-inactivation on complement-bearing immune complexes, complement (co-)factors, and regulators. (**Center**) The depicted scheme represents the initiating steps and essential (co-)factors and regulators of the classical and alternative complement cascade as well as the cleavage pathway of complement factor C3. (**A**–**G**) Concentrations of complement-bearing immune complexes (**A**) C1q-IgG and (**B**) C3d-IgG. (**C**) The concentration and (**D**) activity of complement regulator C1 inhibitor as well as the concentration of complement (co-)factors (**E**) C3c, (**F**) C4, and (**G**) factor B were determined in native sera (blue dots) and after heat-inactivation (red dots). (median of (A) native *n* = 17, heat-inactivated (HI) *n* = 16; (B,E,F,G) *n* =11; (C) native *n* = 15, HI *n* = 14; (D) native *n* = 8, HI *n* = 7; Wilcoxon test; * *p* < 0.05, ** *p* < 0.01, *** *p* < 0.001).

Heat-inactivation of human serum had initially been reported to reduce disturbing effects of complement factors on immunological (such as antibody detection) assays. Nowadays its necessity is controversially discussed. To clarify the impact of heat-inactivation on composition of human sera, we first analyzed classical serum components in native serum before and after heat treatment. Our data show a significant increase in circulating and complement-bearing immune complexes C1q-IgG and C3d-IgG (Figure 1A,B), but also a significant reduction in C1 inhibitor activity and presumably concentration (Figure 1C,D), complement factors C3c and C4 (Figure 1E,F), and alternative pathway co-activator factor B (Figure 1G).

Furthermore, we evaluated immunoglobulin and albumin concentrations. IgG concentration was upregulated, whereas IgE, IgD, and albumin, but not total protein amount, were diminished (Table 1). We suppose that IgG was released by heat from preexisting immune complexes and can only speculate about IgE, IgD, and albumin being denatured or embedded into new complexes. The same might be the reason for reduced high density lipoprotein (HDL). Other metabolic parameters like glucose and triglycerides were not affected by the heat-inactivation process (Table 1).

### 2.2. Heat-Inactivation of Human Serum Impairs Proliferation but Promotes on-Blast Formation of CD4+ T Cells

**Figure 2:**
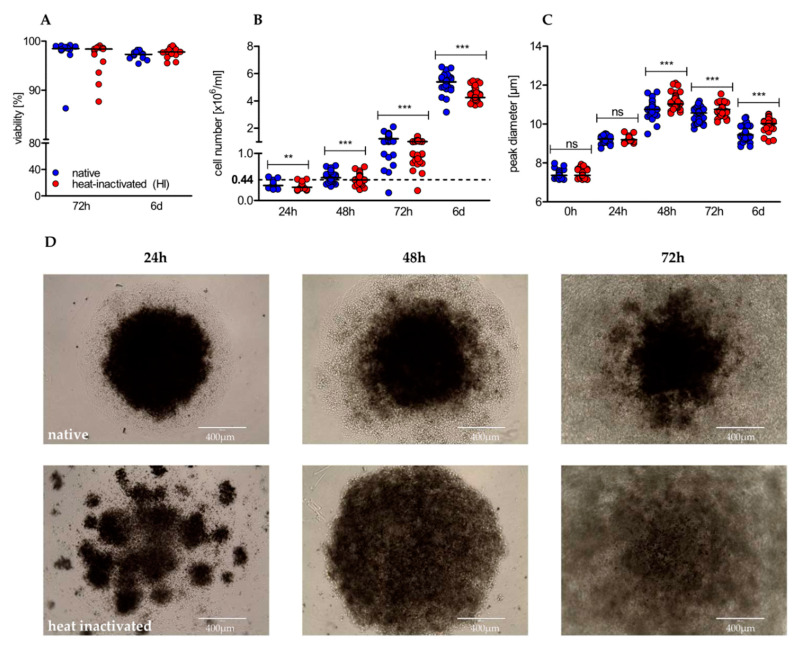
Heat-inactivation of serum barely affects viability, but has significant impact on proliferation, cell growth, and distribution pattern of cultured human CD4+ T cells. (**A**) After 72 h and 6 d of stimulation, viability was determined using the Nicoletti assay by flow cytometry (shown is the median of n = 13 individuals; Wilcoxon test); (**B**) proliferation (shown is the median of 24 h/48 h/72 h/6 d with *n* = 23/43/48/36; Wilcoxon test; ** *p* < 0.01/4, *** *p* < 0.001/4) and (**C**) cell size were analyzed by the CASY system at indicated time points (shown is the median of 0 h/24 h/48 h/72 h/6 d with n = 43/20/40/45/38; Wilcoxon test; * *p* < 0.05/5, ** *p* < 0.01/5, *** *p* < 0.001/5, ns not significant); (**D**) stimulated CD4+ T cells were optically evaluated at indicated time points using an EVOS cell imaging system at a 10-fold magnification. One representative donor is shown.

Next, we analyzed the impact of heat-inactivated (HI) serum in comparison to native serum on viability and proliferation of human CD4+ T cells in the course of stimulation. Heat-inactivation had no impact on the viability of CD4+ T cells (Figure 2A), determined by the Nicoletti assay [47]. Nevertheless, significant differences were observed with regard to proliferation. T cells cultured in native serum proliferated at higher rates at any time point analyzed, resulting in a significantly higher yield after 6 days (Figure 2B). Finally, we analyzed T cell growth taking place in the first phase of activation, referred to as “on blast” formation. In native serum T cells showed a reduced growth, which persisted over time (Figure 2C). Notably, microscopic examination showed a different distribution pattern between native and HI cultured T cells in the early phase of activation. In native cultures T cells formed one circular center, whereas in HI cultures disseminated germinal centers were first observed; these formed a bigger cluster later on (Figure 2D). The difference in on-blast formation and altered distribution pattern and clustering of T cells indicates differences in the first phase of stimulation, characterized by activation-related surface marker expression and by the production and secretion of effector cytokines.

### 2.3. Heat-Inactivation of Human Serum Impairs Proliferation but Promotes on-Blast Formation of CD4+ T Cells

The surface expression of activation-related molecules is regulated dynamically during the stimulation process of CD4+ T cells to meet costimulatory demands and to prevent overstimulation via checkpoints.

**Figure 3:**
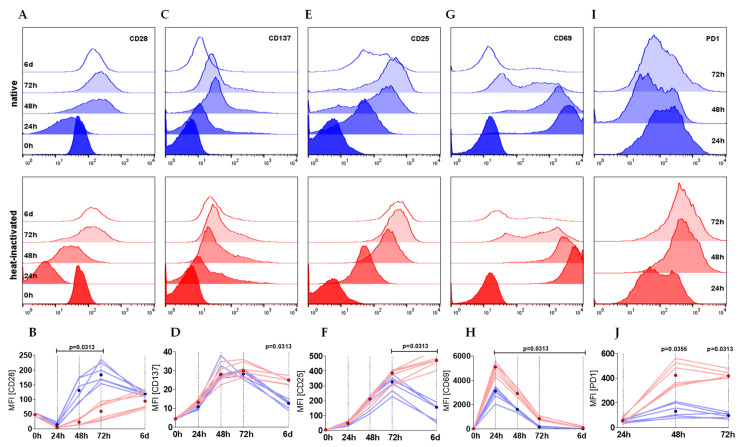
CD4+ T cells cultured in HI serum show a more activated phenotype in terms of surface marker expression. (**A**–**J**) T cells were stimulated either in native or HI serum. CD28 (**A**, **B**), CD137 (**C**, **D**), CD25 (**E**, **F**), CD69 (**G**, **H**), and PD-1 (**I**, **J**) are shown as (**A**, **C**, **E**, **G**, **I**) histogram plots representing a single representative experiment with one donor either cultured in native (blue) and heat-inactivated (red) serum within a time course of 6 days. (**B**, **D**, **F**, **H**, **J**) Depicted graphs show the median fluorescence intensity (MFI) of two individual donors in three different sera (six single experiments each represented by one line) with the dots showing the median of the native (blue) and HI (red) cultures (Wilcoxon test; shown are nominal *p*-values).

CD28, the ligand for CD80 and CD86 expressed on antigen presenting cells, was rapidly downregulated within the first 24 h of stimulation in both culture conditions but afterwards strongly upregulated (Figure 3A,B). The upregulation was accelerated in T cells cultured in native serum correlating with the earlier on-set of proliferation. Moreover, the time-dependent expression pattern was different between the two culture conditions. Whereas CD28 expression was strongly and significantly elevated in the first 3 days and dropped beyond under native culture conditions, HI led to a low but persisting elevation in the course of stimulation. CD28 levels at day six were similar under both conditions (Figure 3A,B). In contrast, TNF receptor family member CD137 (also known as 4-1BB) and interleukin-2 receptor alpha chain CD25 were comparably expressed within the first 72 h of stimulation, however, after 6 days, expression in HI cultures exceeded expression in native cultures (Figure 3C–F). Consistently, the early T cell activation parameter CD69 peaked in HI serum cultured cells after 24 h with a significantly higher median fluorescence intensity (MFI) than expressed in native cultured cells (Figure 3G,H). These observations support the hypothesis of an intensified stimulation of T cells in HI serum. In line, programmed cell death protein 1 (PD-1, CD279), an inhibitory immune checkpoint, was strongly upregulated on T cells cultured in HI serum, but only slightly increased in native cultures (Figure 3I,J).

### 2.4. Heat-Inactivation of Human Serum Promotes Cytokine Secretion, and Increases Metabolic Activity of CD4+ T Cells

We next investigated whether the observed alterations translate into differences in the production of effector cytokines.

**Figure 4:**
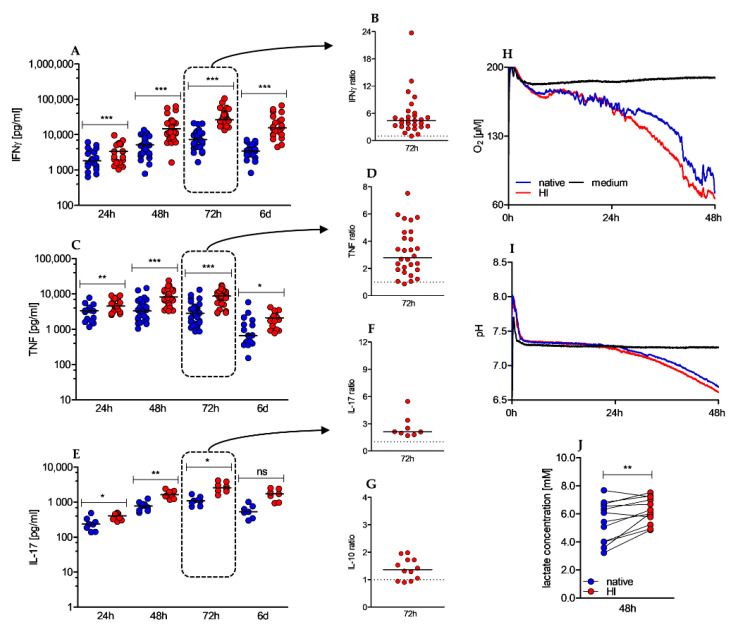
Heat-inactivated serum promotes cytokine secretion and increases O2 consumption and lactate secretion. (**A**–**G**) Cytokine levels in culture supernatants were determined by ELISA after 24 h, 48 h, 72 h, and 6 d. Depicted graphs show absolute values of IFNγ (**A**), TNF (**C**) and IL-17 (**E**) over time and the ratio of IFNγ (**B**), TNF (D), IL-17 (**F**), and IL-10 (**G**) in HI to native serum conditions after 72 h of stimulation; (shown is the median, IFNγ 24 h *n* = 23, 48 h *n* = 43, 72 h *n* = 29 (native)/28 (HI) and 6 d *n* = 21; TNF 24 h *n* = 17; 48 h *n* = 37 (native)/36 (HI); 72 h *n* = 28; 6 d *n* = 17; IL-17 24 h *n* = 8; 48 h *n* = 11; 72 h *n* = 8; 6 d *n* = 7; IL-10 72 h *n* = 12, Wilcoxon test, * *p* < 0.05/4; ** *p* < 0.01/4; *** *p* < 0.001/4, ns not significant). To analyze O2 consumption (**H**) and extracellular pH levels (**I**), (H–I) 0.8 × 106 CD4+ T cells were anti-CD3/CD28 bead stimulated for 48 h in a PreSense Sensor Dish plate. One representative experiment is shown. (**J**) Supernatants of stimulated CD4+ T cells were withdrawn after 48 h of stimulation to enzymatically measure extracellular lactate concentrations in native and HI serum cultures (*n* = 12, Wilcoxon test; ** *p* < 0.01).

Besides cellular cytotoxicity, effector functions of human CD4+ T cells comprise secretion of pro- and anti-inflammatory cytokines. In Th1 and Th17 responses, IFNγ, TNF, and IL-17 assume these tasks. We observed a significantly elevated cytokine secretion in HI versus native cultured conditions (Figure 4A–F). Remarkably, this effect was already observed within the first 24 h and sustained over the whole observation period. To evaluate whether HI of the serum affects cytokine secretion in general or is restricted to pro-inflammatory cytokines, we also determined the concentration of the anti-inflammatory cytokine IL-10, which was found to be significantly elevated after 72 h, however to a much lesser extent (Figure 4G). Activation of T cells is an energy-demanding process. In human T cells, oxidative phosphorylation (OXPHOS) and anaerobic glycolysis (“Warburg effect”) deliver the energy carrier adenosine triphosphate (ATP). While OXPHOS is characterized by oxygen consumption, anaerobic glycolysis produces lactic acid resulting in increased extracellular lactate and acidification. In our analysis we found elevated oxygen consumption (Figure 4H), slightly increased acidification rates (Figure 4I), and significantly elevated extracellular lactate concentrations (Figure 4J) in heat-inactivated cell cultures. As these observations were made within the first 48 h, a profound impact of proliferation can be almost excluded as proliferation starts beyond that time. Taken together, HI of the serum results in strongly elevated cytokine secretion accompanied by increased metabolic activity. 

### 2.5. Effects of Heat-Inactivation Are Partially Reverted by c1 Inhibitor and Mimicked by Anti-C1q Antibody Supplementation

**Figure 5:**
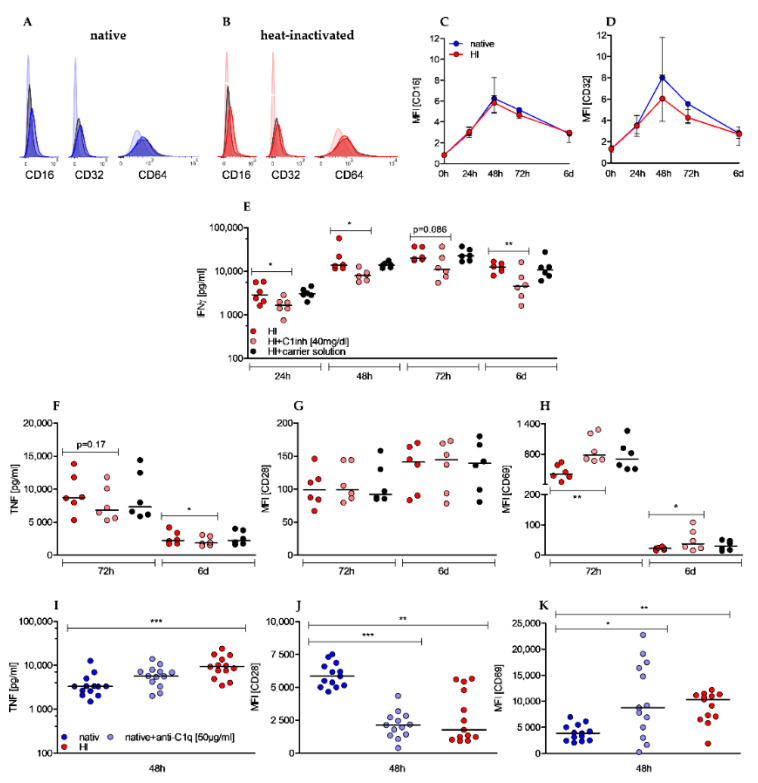
Effects of heat-inactivation are not related to Fcγ receptors II (CD32) and III (CD16) expression but are partially reverted by C1 inhibitor and mimicked by anti-C1q antibody supplementation. (**A**,**B**) Histogram plots display expression of CD16, CD32, and CD64 (opaque colors) compared to isotype controls (black) and unstained cells (transparent color) in native (A) and HI cultured cells (B) after 48 h of stimulation. One representative experiment is shown. (**C**,**D**) ΔMFI (difference of MFI antibody and MFI isotype) of CD16 (**C**) and CD32 (**D**) is depicted in a time-dependent manner. Shown is the median ± interquartile range of *n* = 4 (24 h/48 h/72 h/6 d) and *n* = 2 (0 h). (**E**–**H**) C1 inhibitor was added to T cells cultured with heat inactivated serum. In the presence of C1 inhibitor IFNγ (**E**), TNF (**F**), CD28 (**G**), and CD69 (**H**) were analyzed at indicated time points. (Shown is the median of *n* = 6, each dot represents one donor, Friedman test, post-hoc Dunn’s, * *p* < 0.05, ** *p* < 0.01). (**I**–**K**) Anti-C1q antibody was added to T cells cultured with native sera. TNF secretion (**I**) was measured by ELISA and CD28 (**J**) and CD69 (**K**) expression was analyzed by flow cytometry at indicated time points. (Shown is the median of *n* = 13; Friedman test, post-hoc Dunn’s, * *p* < 0.05 ** *p* < 0.01; *** *p* < 0.001).

Antigen–antibody complexes (immune complexes) are potent immune stimulators and interaction with immune cells is facilitated by the fragment crystallizable (Fc) portion of surfacing IgG antibodies (Fcγ). To examine whether IgG antibody receptors contribute to the observed effects by HI sera we analyzed the surface expression of Fcγ receptors III (CD16), II (CD32), and I (CD64) on CD4+ T cells during stimulation. Under both culture conditions expression of CD16 and CD32 was low in unstimulated cells and only slightly upregulated to peak at 48 h before declining again. In contrast, CD64 was not expressed above isotype levels (Figure 5A,B). Although an expression of FcγR II and III was clearly detectable, the extent was only marginal and not different between T cells cultured in native serum or HI serum, thereby hardly explaining the strong effects of heat-inactivation (Figure 5C,D). As heat-inactivation also significantly diminished activity and concentration of C1 inhibitor (C1inh), we examined whether supplementation of human C1 inhibitor to heat-inactivated sera was able to revert the observed pro-stimulatory effects. The addition of C1 inhibitor could neither revert the effects on proliferation nor on growth (data not shown), however, we observed an effect on IFNγ levels. In native serum we measured 27% of the IFNγ levels compared to HI serum after 72 h and the addition of the C1 inhibitor reduced IFNγ secretion to 54% (Figure 5E). Therefore, the C1 inhibitor accounted for 50% of the lower IFNγ levels in native serum. The impact of the C1 inhibitor on TNF secretion was weaker and time-delayed as it was only detectable beyond 48 h of stimulation (Figure 5F). Furthermore, the effect of heat-inactivation on CD28 (Figure 5G) and CD69 (Figure 5H) cannot be explained by the impact of C1 inhibitor as the addition resulted in a comparable or even more pronounced upregulation. Besides a diminished C1 inhibitor concentration, heat-inactivated sera were also characterized by an increased concentration of C1q-binding immune complexes. In order to induce complex formation with C1q to mimic the effect of heat-inactivation, we examined whether addition of anti-C1q antibodies to native sera had comparably promoting effects on T cell activation. While cell number, cell growth, and IFNγ secretion were not affected during a 48 h stimulation period, a clear effect on TNF secretion was observed although not significant (Figure 5D).

Additionally, compared to T cells in native sera, expression of costimulatory receptor CD28 was significantly reduced (Figure 5J) and early activation marker CD69 was increasingly expressed (Figure 5K), which resembled the effect of HI sera. These results indicate a positive effect of complex-forming anti-C1q antibody on stimulation of CD4+ T cells. As summarized in Table 2, sole addition of C1 inhibitor to HI sera or anti-C1q antibody to native sera only partially reverted or imitated the impacts of heat-inactivation on activation of human T cells.

## 3. Discussion

Heat-inactivation of sera is a frequently used method to improve culture conditions for different types of cells based on the hypothesis that complement factors impair cell proliferation and function. In our measurements, stimulation of human CD4+ T cells in heat-inactivated human sera showed an overall increased activity profile with stronger increase in cell size (“on-blast formation”), albeit at the expense of a persistently reduced cell number. The costimulatory receptor CD28 was sequentially down- and the inhibitory receptor PD-1 upregulated in HI sera likely preventing overstimulation [48,49] and the increased expression of the early activation marker CD69 underlined the activated state of T cells in heat-inactivated sera [50,51]. The effector function of CD4+ T cells was also enhanced by heat-inactivated sera as secretion of the Th1 cytokines IFNγ and TNF, but also of IL-17 and IL-10, was strongly increased. Anaerobic glycolysis and mitochondrial respiration, both of which are strengthened by heat-inactivated sera, provide energy and biomass to activated T cells [52,53] and are tightly intertwined with T cell effector functions [54,55]. In our study, heat-inactivated serum (Figure 1) resembled the serum of patients suffering from systemic lupus erythematosus—an excess of C1q-bearing, circulating immune complexes (CICs) [56,57] and a lack of the classical pathway regulator C1 inhibitor (C1inh) [58,59], which through activation and simultaneous lack of inhibition results in consumption-related hypocomplementemia [60]. C1q-bearing immune complexes interact with T cells via surface C1q receptors (C1qR) [40] and induce a CD25-low phenotype that secrets IFNγ and TNF even without TCR signaling [39]. These observations have also been made when C1q is bound by anti-C1q instead of immune complexes [39]. In our study, addition of anti-C1q antibody to native sera pushed T cells towards the HI phenotype regarding expression of activation-related surface markers CD28 and CD69 and to a minor extent to TNF secretion, whereas proliferation, cell growth, and IFNγ secretion did not meet the heat effects, and expression of CD25 showed diametrically opposed results. Although several studies have examined the influence of CICs [39,40,42] and complement factor C3 [27,29,34,35] on the activation of T cells, only little is known about the effects of C1 inhibitor on human T cells. Heat-inactivation reduced the concentration and activity of the C1 inhibitor in the serum almost completely. In addition to the classical complement pathway [61,62,63], serine proteinase (Serpin) C1 inhibitor also regulates the coagulation [64,65] and kallikrein cascade [66,67] and thus acts as a systemic regulator of inflammatory reactions. By adding physiological concentrations of the human C1 inhibitor to the HI serum, we examined the extent to which the observed effects could be attributed to the heat-mediated C1 inhibitor deficiency. While the effects on proliferation, cell growth, and surface markers were negligible (Table 2), a significant reduction in the production of Th1 cytokines IFNγ and TNF was shown indicating that the pronounced cytokine production in the presence of heat-inactivated serum was partially based on C1 inhibitor deficiency. These results are in diametrical contrast to studies from the 1990s showing a reduction in proliferation with increased IFNγ secretion under the influence of C1 inhibitor [68,69]. However, the studies were performed with (1) unselected mononuclear cells and mixed leukocyte reactions, respectively, and (2) lower C1 inhibitor concentrations of between 10 to 400 µg/mL. Jiang et al. showed that C1 inhibitor is also a regulator of the alternative complement pathway and mediates this effect through strong binding to the C3b. However, this effect was only observed in immobilized C3b and data on binding of C1 inhibitor to liberated C3b are missing [70]. C3a and C3b contribute decisively to the initiation of a Th1 response in CD4+ T cells and promote cell cycle progression and increased secretion of the Th1 cytokines IFNγ and TNF. Thus, C3-mediated co-stimulation results in a T cell phenotype that is similar to that of HI-cultured CD4+ T cells. Based on these findings we speculated that the C1 inhibitor could bind to C3b released during TCR stimulation and prevent it from its co-stimulatory interaction with CD46. A C1 inhibitor deficiency caused by heat-inactivation would release this complement-mediated immunological brake and lead to an increased immune response, which in turn could be reversed by adding the C1 inhibitor. Consistent with these considerations, manifold studies prove the co-occurrence of hereditary C1 inhibitor deficiency (appearing as hereditary angioedema) with autoimmune diseases such as systemic lupus erythematosus and endocrine autoimmune disorders [71,72,73].

Activation and infiltration by T cells contribute directly to the fight against and prognosis of tumor diseases [74,75]. Numerous immune escape mechanisms limit this immune response and thus contribute to the progression of the disease. Recent work from H. Redebrandt’s group showed that C1 inhibitor is produced by glioblastomas [76] and pancreatic carcinomas [77]. The addition of anti-C1 inhibitor antibodies led to beneficial effects on overall survival in an animal model. The authors speculate that the overexpression protects the tumor by inhibiting the classical complement pathway. Based on our results, direct interference with tumor-infiltrating CD4+ T cells should also be considered and C1 inhibitor secretion by solid tumors could thus represent a new immune escape mechanism.

In summary, we were able to show that heat-inactivation of serum leads to C1 inhibitor deficiency and an excess of C1q-binding immune complexes resulting in clearly improved human T cell function. By adding C1 inhibitor in physiological concentrations, the stimulating effect on IFNγ and TNF production could be partially reversed, whereas effects of HI on surface markers were mimicked by complex-forming anti-C1q antibody. In contrast, effects on proliferation and cell growth seem to relate to other serum factors.

## 4. Material and Methods

### 4.1. Heat-Inactivation of Human Sera

A total of 10 single and four pooled human sera (BRK, Bavarian Red Cross, Munich, Germany) were heat-inactivated in a pre-heated water bath at 56 °C for 30 min (according to freely available culture protocols) and subsequently stored at −20 °C until used.

### 4.2. Determination of Heat-Induced Changes in Serum Composition

Heat-induced changes in human sera were analyzed at the Department of Clinical Chemistry, University Hospital Regensburg, Germany (Table 3).

C1 inhibitor activity was determined by applying an enzymatic immunoassay, administering biotinylated C1s as a substrate. With this assay C1 inhibitor activity/functional free C1 inhibitor in the serum is quantified and compared to a standard sample, values above 68% are regarded as normal activity in the serum. Protein concentration was measured via nephelometry, administering an N antiserum against C1 inhibitor.

### 4.3. Human T Cell Isolation and Culture

Human T cells were isolated from PBMCs of healthy donors after leukapheresis or directly from leukocyte reduction system cones followed by density gradient centrifugation over Ficoll/Hypaque. CD4+ T cells were enriched by Miltenyi magnetic bead separation (Miltenyi Biotec GmbH, Bergisch Gladbach, Germany). The T cell purity was ˃98% as determined by CD4 (PE, RPA-T4, BD Bioscience, Heidelberg, Germany) expression by flow cytometry. All participants provided written informed consent and the study was approved by the local ethics committee (vote numbers 13-101-0240 and 13-101-0238).

T cells were stored overnight in a 24-well flat bottom plate at a concentration of 10 × 10^6^ cells/mL in T cell medium consisting of RPMI 1640 (Thermo Fisher, Waltham, MA, USA, 31870-025) supplemented with 10% either heat-inactivated (HI) or not heat-inactivated (native) human serum (BRK, Bavarian Red Cross), L-glutamine (2 mM, PAN-Biotech, Aidenbach, Germany, P04-80100), essential vitamins and non-essential amino acids (1×, Thermo Fisher, 11120037 and 11140035), pyruvate (1 mM Thermo Fisher, 11360039), β-mercaptoethanol (50 µM, Thermo Fisher, 31350010), and penicillin and streptomycin (50 IU/mL, Thermo Fisher, 15140122) in a humidified atmosphere (5% CO2, 95% air) at 37 °C in a Heraeus incubator (Thermo Fisher).

Subsequently, 0.1 × 10^6^ T cells were cultured in T cell medium supplemented with 25 IU/mL of recombinant human IL-2 (PeproTech, Rocky Hill, NJ, USA, 200-02) in 96-well U-bottom plates and stimulated with anti-CD3/CD28 dynabeads (Thermo Fisher) at a cell to bead ratio of 1:1. After 72 h, stimulated T cells were split and medium was replenished to the initial well volume of 225 µL. After 24, 48, 72 h, and 6 days supernatants were withdrawn for further analysis and cell size and cell number were measured using the CASY system (Roche Innovatis, Bielefeld, Germany). In addition, cells were examined cytomorphologically and with regard to the distribution pattern in the cell culture plate with an EVOS cell imaging system (OLS, Bremen, Germany).

### 4.4. Flow Cytometry

After 24 h, 48 h, 72 h, and 6 d cultured cells were harvested, magnetically separated from stimulation beads and washed with FACS buffer (phosphate buffered saline with 2% fetal calf serum). Viability was determined by flow cytometry after 72 h and 6 d using Nicoletti buffer according to the protocol described elsewhere [47]. Surface markers were stained with anti-human antibodies against CD28 (CD28.2, BD, Heidelberg, Germany), CD137 (4B4, eBioscience, Frankfurt a.M., Germany), CD25 (M-A251, BD), CD69 (FN50, BD), CD16 (3G8, BD Bioscience), CD32 (FUN-2, BioLegend, San Diego, CA, USA), CD64 (10.1, BD Bioscience), CD152 (BN13, BD Bioscience), and CD279 (EH12.2H7, BioLegend). Isotypes and quiescent cells were stained as negative controls. The data were recorded on a FACS Calibur cytometer or an LSRFortessa flow cytometer (both BD), and analyzed using FlowJo software (Tree Star, Ashland, OR, USA).

### 4.5. Determination of Cytokines

The concentration of IFNγ, TNF, IL-17, and IL-10 in cell culture supernatants was determined after 24 h, 48 h, 72 h, and 6 d by commercially available enzyme-linked immunosorbent assays (R&D Systems, Minneapolis, MN, USA) according to the manufacturer’s protocol.

### 4.6. Monitoring of Oxygen Consumption and pH Development and Determination of Lactate Secretion

Cellular oxygen consumption and pH changes in culture medium were determined non-invasively by the PreSens technology (PreSens Precision Sensing GmbH, Regensburg, Germany). For this, 0.8 × 10^6^ T cells with anti-CD3/CD28 dynabeads (with a cell to bead ratio of 1:1, Thermo Fisher Scientific) were seeded in 24-well Oxodish^®^ OD24 or Hydrodish^®^ HD24 plates without fixation in 1 mL T cell medium supplemented either with heat-inactivated or native human serum under cell culture conditions for the indicated period of time and measurements were taken at 60 s intervals.

Lactate concentration was determined using a Roche cobas^®^ pro and specific reagents (Roche Diagnostics, Mannheim, Germany) at the Department of Clinical Chemistry, University Clinic, Regensburg, Germany.

### 4.7. Supplementation of C1 Inhibitor to Heat Inactivated Sera

The human C1 inhibitor (C1inh) Berinert was purchased from CSL Behring GmbH, Marburg. According to the analysis certificate (Batch number P100127740), the solution obtained by resuspension contained 8 mg/mL protein with a 94% purity. A concentration of 40 mg/dL (corresponding to 11.25 µL/well) was added to the cell cultures reflecting the upper limit of physiologic concentration. In order to exclude interfering effects, the carrier solution was prepared according to the manufacturer’s instructions (glycine 9.6 mg/mL, sodium chloride 8 mg/mL, sodium citrate 2.8 mg/mL), filtered using a 0.2 µm filter, and added at 11.25 µL/well to a control series.

### 4.8. Modification of Native Sera by Anti-human Anti-C1q Antibody

Anti-C1q antibody was administered according to Jiang et al., slightly modified [39]. In order to induce complex formation with serum contained C1q and therewith imitate the effect of heat-inactivation, we added a final concentration of 50 µg/mL anti-human anti-C1q antibody (NB100-64420, Novus Biologicals, Centennial, CO, USA) to cells cultured in native sera.

### 4.9. Statistics and Design

Statistical parameters including exact value of n and statistical significance are reported in the figure legends. Statistical analysis was performed with the GraphPad Prism software version 5 (GraphPad Software, Inc., San Diego, CA, USA). Data were judged to be statistically significant when *p* < 0.05 by Wilcoxon signed-rank test for comparison of paired, non-parametric groups, significance levels were corrected for multiple testing by the Bonferroni correction of the significance levels, i.e., the p value is divided by the number of tests performed (e.g., * *p* < 0.05/4, 4 is the number of tests performed and the new *p* value for * *p* < 0.0125; stated in the figure legends). For the comparison of more than two groups the Friedman Test was applied as data could not be tested for normality distribution, post-hoc analysis were performed with the Dunn’s test.

## Figures and Tables

**Table 1:** Effects of serum heat-inactivation on immunoglobulins, proteins, glucose, and lipids. (mean ± SEM of *n* = 9, except for IgE, where native *n* = 9, HI *n* = 8, and glucose *n* = 4; Wilcoxon test; * *p* < 0.05, ** *p* < 0.01, ns not significant).

	Native Sera Mean (±SEM)	Heat-Inactivated Sera Mean (±SEM)	Significance Level
IgG	1146 (58.14)	1369 (66.43)	**
IgE	0.0764 (0.0472)	0.0017 (0.0006)	**
IgD	2.622 (0.562)	0.510 (0.164)	**
Albumin	4254 (112.6)	3797 (135.2)	*
Total protein	7266 (138.1)	7282 (187.5)	ns
Glucose	71.25 (5.170)	71.25 (5.218)	ns
Cholesterol	196.3 (9.107)	196.2 (9.646)	ns
−HDL	56.89 (3.553)	49.22 (3.582)	*
−LDL	109.3 (9.042)	110.7 (9.006)	ns
−VLDL	30.11 (3.839)	36.33 (4.752)	ns
Triglyceride	122.4 (10.29)	120.9 (9.945)	ns

*Abbreviations:* HDL, high density lipoprotein; LDL, low density lipoprotein; and VLDL, very low density lipoprotein.

**Table 2:** Schematic illustration of the effects of HI, HI + C1inh, and native + anti-C1q on human CD4+ T cells compared to cells in native serum. Effects are analyzed after 72 h (HI, HI + C1inh) and 48 h (native + anti-C1q), respectively. Each arrow represents a significantly increased (↑), significantly decreased (↓) effect or no significant effect (↔). Effects being also induced by the carrier solution are depicted as no significant effect.

48/72 h	+anti-C1q [50µg/ml]	native	HI	+C1inh [40 mg/dL]
**cell number**	↔		↓	↔
**peak diameter**	↔		↑	↔
**IFNγ**	↔		↑	↓
**TNF**	(↑)		↑	↓
**CD28**	↓		↓	↔
**CD69**	↑		↑	↑
**CD137**	↔		↔	↔
**CD25**	↓		↑	↔

**Table 3:** Tests, equipment, and methods used for determination of heat-induced changes.

Parameter	Company	Device	Method
CIC C1q-IgG	Human GmbH (Wiesbaden, Germany)		1
CIC C3d-IgG	Human GmbH		1
C1 inhibitor activity	TECO medical (Sissach, Swiss)		1
C3 activator	The Binding Site GmbH (Schwetzingen, Germany)		2
IgE	Roche Diagnostics (Mannheim, Germany)	Cobas e411	3
IgG	Siemens Healthcare Diagnostics (Erlangen, Germany)	Dimension Vista 1500	4
IgD	The Binding Site GmbH	BN ProSpec	4
C1 inhibitor concentration	Siemens Healthcare	BN ProSpec	4
C3c	Siemens Healthcare Diagnostics	Dimension Vista 1500	4
C3c	Roche Diagnostics (since 01/2020)	Cobas pro	5
C4	Siemens Healthcare Diagnostics	Dimension Vista 1500	4
C4	Roche Diagnostics (since 01/2020)	Cobas pro	5
Albumin	Siemens Healthcare Diagnostics	Dimension Vista 1500	6
Electrophoresis	Sebia	Capillaries	7
Total protein	Siemens Healthcare Diagnostics	Dimension Vista 1500	6
Glucose	Siemens Healthcare Diagnostics	Dimension Vista 1500	6
Cholesterol	Siemens Healthcare Diagnostics	Dimension Vista 1500	6
Triglyceride	Siemens Healthcare Diagnostics	Dimension Vista 1500	6
HDL-cholesterol	Siemens Healthcare Diagnostics	Dimension Vista 1500	6
LDL-cholesterol	Siemens Healthcare Diagnostics	Dimension Vista 1500	6
VLDL-cholesterol		Calculated	

*Methods:* 1, ELISA; 2, immunodiffusions; 3, electrochemiluminescence; 4, nephelometry; 5, immunoturbidimetry; 6, photometry; and 7, capillary electrophoresis
